# Development of a Mobile Phone App to Support Self-Monitoring of Emotional Well-Being: A Mental Health Digital Innovation

**DOI:** 10.2196/mental.6202

**Published:** 2016-11-23

**Authors:** Nikki Rickard, Hussain-Abdulah Arjmand, David Bakker, Elizabeth Seabrook

**Affiliations:** ^1^ Emotion and Well-being Research Unit School of Psychological Sciences Monash University Clayton Australia; ^2^ Centre for Positive Psychology Melbourne Graduate School of Education University of Melbourne Melbourne Australia

**Keywords:** eHealth, emotions, mental health, mobile phone, feedback

## Abstract

**Background:**

Emotional well-being is a primary component of mental health and well-being. Monitoring changes in emotional state daily over extended periods is, however, difficult using traditional methodologies. Providing mental health support is also challenging when approximately only 1 in 2 people with mental health issues seek professional help. Mobile phone technology offers a sustainable means of enhancing self-management of emotional well-being.

**Objective:**

This paper aims to describe the development of a mobile phone tool designed to monitor emotional changes in a natural everyday context and in real time.

**Methods:**

This evidence-informed mobile phone app monitors emotional mental health and well-being, and it provides links to mental health organization websites and resources. The app obtains data via self-report psychological questionnaires, experience sampling methodology (ESM), and automated behavioral data collection.

**Results:**

Feedback from 11 individuals (age range 16-52 years; 4 males, 7 females), who tested the app over 30 days, confirmed via survey and focus group methods that the app was functional and usable.

**Conclusions:**

Recommendations for future researchers and developers of mental health apps to be used for research are also presented. The methodology described in this paper offers a powerful tool for a range of potential mental health research studies and provides a valuable standard against which development of future mental health apps should be considered.

## Introduction

### Background

Emotional well-being is broadly defined [1] as, “a positive sense of well-being and an underlying belief in our own and others’ dignity and worth” by the Mental Health Foundation (p. 8). Consistent with dual models of well-being, it encompasses both positive functioning (happiness, a sense of control and self-efficacy, and social connectedness) and an absence of stress and depression [2,3]. Monitoring changes in emotional well-being is fundamental to mental health, with increases in emotional well-being associated with resilience, creative thinking, social connectivity, and physical health [4-9]. In contrast, significant and sustained decreases in emotional well-being are associated with the development of affective disorders such as depression and anxiety, and reduced physical health [4,5,7].

Monitoring for such changes is crucial for early detection of mental health problems. Rapid response to early risk indicators is one of the key predictors of better health outcomes, enabling preventative health approaches to be initiated early [10]. Regular monitoring of emotional health indices is therefore recommended by various national guidelines [11,12]. In practice, however, it remains difficult for clinicians or professional mental health service providers to obtain frequent monitoring in real time [13,14]. A priority challenge facing the health care system is to achieve practicable and sustainable means of supporting self-management of health and well-being. Self-monitoring is a particularly attractive goal for mental health care, given that many individuals with mental health needs do not seek professional health care support [15-17]. In addition, self-monitoring may develop an individual’s insight into their need to seek help. In particular, young people consistently indicate that they prefer nonprofessional or self-managed strategies for addressing mental health issues [18,19]. Obtaining temporally sensitive (eg, daily) information on significant changes in emotional state has the potential to profoundly improve the capacity to promote emotional health [12].

Experience sampling methodologies (ESMs), or ecological momentary assessments, involve the systematic collection of self-report data from individuals at multiple time points throughout their everyday lives [20]. ESMs have been used to monitor changes in affective state, and to predict mental health with success to a certain extent [21,22]. In particular, the variability in emotional state over time provides more substantial information for understanding the causes and nature of psychopathology than do cross-sectional “snapshot” assessments. For example, when sampled multiple times a day for 6 days, negative affect was found to vary more in patients diagnosed with major depressive disorder than that in controls across the day [23]. ESM assessments in individuals diagnosed with panic disorder also revealed that the expectation of a panic attack was a significant precursor for the occurrence of a panic attack [24]. Ben-Zeev et al [25] also found that patients diagnosed with a major depressive disorder retrospectively reported higher levels of symptoms relating to anhedonia, suicidality, and sadness than captured in their ESM reports, highlighting the biases of traditional survey methods. To date, however, it has been methodologically difficult and obtrusive to obtain temporally regular and precise measures of emotional state [21]. The resources required to obtain such information repeatedly over lengthy time frames have made such an intensive monitoring prohibitive. In addition, the use of palm pilots and pagers (which were never as familiar to users as mobile phones have become) to prompt users for this information can be intrusive, and makes it less likely that users will continue to use this form of monitoring for extended periods [26].

Mobile phone technology offers an unprecedented opportunity to unobtrusively track everyday behavior and changes in emotional state, all in real time [27,28]. Mobile phone health tools also offer the potential of immediate response to the outcome of this monitoring via delivery of mental health information contingent on changes in real-time emotional state [29]. This technology has not yet been fully leveraged for these purposes, despite mobile phones being one of the few pieces of technology that most people carry on their person every day [30]. This pervasiveness means that mobile phones offer a highly natural and regular means by which information on emotional state could be obtained. Mobile phones now penetrate 77%, 72%, and 68% of the Australian, US, and UK population, respectively [31], and are a cost-effective means of seeking help for mental health issues that may overcome socioeconomic and geographic boundaries [32,33].

Mobile phone health technology holds great potential for facilitating the management of emotional health through its ability to deliver flexible, user-oriented intervention and self-management tools; a feature particularly relevant for young people who often report fear of stigma associated with seeking professional services for sensitive mental health issues [34,35]. In a 2010 study, 76% of an Australian sample reported being interested in using mobile phones to monitor and manage their own mental health [32]. A large number of mobile phone apps are currently available that claim to promote mental health and well-being [36,37] and a subset of these also attempt to track mood or emotional state over time. However, empirical support for the efficacy of these apps is extremely limited [36]. For instance, in a systematic review of 5464 mental health app abstracts, less than 5 apps were found to have experimental evidence [37]. In addition, a few have capitalized on the benefits enabled by the mobile phone technology such as experience sampling and automated data collection in identifying and evaluating potential time-sensitive behavioral indicators of mental health change [36].

Of the mobile phone mental health programs that have utilized ESM to track mood over time, several favorable outcomes have been reported. For example, Reid et al [28,38] found that the majority of their adolescent sample using the mobile phone-based mental health app, *mobiletype*, completed their self-assessments, and that the use of the app increased the practitioners’ understanding of their patients’ mental health. Harrison et al [29] reported that the use of the mobile phone accessed Web-based cognitive behavioral therapy (CBT) course *MyCompass* for 6 weeks significantly reduced symptoms of depression and anxiety and improved self-efficacy. One of the barriers to sustainability of user engagement in such programs, however, is that they require extensive voluntary input from the user. When evaluated, a common theme is initial compliance, followed by high dropout and poor self-reporting rates (eg, less than 10% of the sample trialing *MyCompass* reported using it every day) [29]. Reasons for discontinued use include problems understanding how to use the program, invasiveness of the questions, the need for repetitive completion of questionnaires, insufficient personalization of the mental health advice, and little motivation to engage with the program [28,29].

An innovative way to meet this challenge is to monitor indices of emotional health using methods that require minimal insight or subjective report from the user. Mobile phones contain a range of embedded sensors and features, including accelerometers and global positioning systems and apps, which can automatically record information about a user’s behavior [39]. Two recent studies have obtained a combination of data from mobile phones in an attempt to predict participants’ self-reported mood. LiKamWa et al [40] found that up to 93% of mood scores were accurately predicted by social activity, physical activity, and general mobile phone use data collected from mobile phones. Asselbergs et al [41] attempted to predict self-reported mood of 27 participants from metadata of 6 mobile phone indices (phone calls, text messages, screen time, app usage, accelerometer, and phone camera events). Although the accuracy of the models was no greater than models obtained without mobile phone data, the methodology was demonstrated to be technically feasible and to hold promise. The authors recommended that inclusion of more meaningful or relevant features from mobile phone data may be the key to improving prediction.

Interestingly, young people use mobile phones for music listening, fitness, and social networking more than any other demographic [42], and these are among the most effective strategies for optimizing emotional health [43-46]. For example, the frequency of app-switching and the content of social network messages were found to predict depression [43] even prior to its onset [47]. Music listening patterns also appear to predict emotional health [48-50] and given that approximately two thirds of music listening by young people is via mobile devices such as mobile phones [31], it is surprising that relatively few apps have attempted to use music for this purpose [27]. Vocal expression too has been found to be a useful index of emotional state [51,52]. Short voice samples have been found to demonstrate 70% accuracy for simple affect recognition [53]. Monitoring a combination of behavioral indices such as physical activity, online social interactions, and music choices therefore offers a promising means of nonintrusive but sensitive assessment of affective state. Advances in statistical methods available through machine learning also enable powerful analysis of this more complex level of individualized multilevel modeling [52,54].

Another limitation of most mental health apps currently available is that they tend to simplify the emotional well-being spectrum, with positive and negative affect anchors on a unidimensional rating scale. Contemporary conceptualizations of well-being however clearly show that optimal “emotional health and well-being” does not emerge from an absence of affective disorder alone, but also requires a state of positive functioning [2,55,56]. Although positive and negative emotional functioning are correlated, there is substantial evidence that they are orthogonal constructs [57]. Mobile phone technology that differentiates the quadrants created by categorizing according to mental illness or languishing and mental health or flourishing [3,55] is therefore encouraged.

### Objective

In this paper, we capitalized on the extraordinary role that mobile phones play in people’s lives to develop a tool that has the potential to significantly extend the understanding of emotional health and well-being. The aim of this paper was to describe the design of the mobile phone app, *MoodPrism*, which was developed to monitor emotional well-being in context and in real time, and provide personalized feedback on the full spectrum of emotional well-being. The paper describes in detail the design and data collection functions of the app, which were incorporated to address major challenges for mental health research and practice, and presents feedback from a small sample of trial users (beta-testers), which tested the functionality and usability of the app.

## Methods

### Design and Development of the App

*MoodPrism* was designed and developed in collaboration with a commercial digital creation studio, Two Bulls (Melbourne, Australia). The app was prepared for both the iOS and Android mobile phone platforms and was distributed by the Web-based Apple and Google Play stores, respectively. The term “*MoodPrism*” was selected to reflect its primary purpose of collecting emotional state data across the entire spectrum of emotional health and well-being and converting this into an array of color-coded feedback to the user.

The development of *MoodPrism* involved designing 3 different methods of data collection within the software: (1) automated monitoring of selected online behavior, (2) experience sampling of emotional well-being self-reports, and (3) psychological assessment questionnaires. automated monitoring of selected online behavior, experience sampling of emotional well-being self-reports, and psychological assessment questionnaires. This triangulation of data collection is considered crucial for advancing the measurement of emotional state [58]. As part of the sign-up procedure to the app, permissions for sensitive data had to be obtained. Incentives to continue collecting data over an extended period were also generated.

The development of *MoodPrism* was completed in March 2015. The required forms of data collection were achieved by developing a suite of app components, which were then collated into a cohesive app. The outcomes of this development process are described in the following.

#### Sign Up

As part of the sign-up procedure for the app, options were offered to users to provide the app with access to social networking and music apps as well as general (postcode) location. These data were then collected continuously and without the need for user input over the month’s research period. After sign up and consent procedures, *MoodPrism* administered the initial surveys that could be completed in multiple sittings and required 30-60 min in total to complete. The participants were then requested to use the app for at least thirty days, during which they would be prompted daily to answer a set of short questions, and weekly to complete a short audio recording. If they were unable to respond to daily prompts, *MoodPrism* advised they could complete them at a time of their convenience till midnight that day, or alternatively to ignore them. At the end of the 30 days, users were invited to complete a final set of surveys, which in total required 15-30 min to complete.

Users were incentivized to continue using *MoodPrism* through 3 strategies. First, daily mood and mental health feedback was provided to the user, with additional feedback unlocked after sustained use (Multimedia Appendix 2). This promoted engagement by rewarding users and encouraging feelings of achievement, adhering to principles of gamification [59], which is recommended in mental health apps [36]. Second, completion of daily reports as well as the final surveys generated entries into a draw for 1 of the 4 $AU100 (approximately US $75) gift vouchers. Third, users were informed that their data were contributing to research into the value of mobile phone apps for monitoring mental health and well-being.

#### Automated Monitoring

*MoodPrism* acted as a portal for data accessed via several mobile phone sensors and apps. Two validated predictors of emotional state change were targeted: music use and web-based social network site activity. As a part of the sign-up process, users were invited to give permission for the app to access Facebook, Twitter, the user’s music library, and location (postcode only).

Facebook, Twitter, and music use data were collected once every 24 h, and the information collected is provided in Multimedia Appendix 1. Data were accessed from Facebook and Twitter through their relevant application programming interfaces (APIs). This allows third-party access to selected data collected by both Facebook and Twitter. Facebook and Twitter content was analyzed automatically and locally on the user’s phone using several linguistic dictionaries from the Linguistic Inquiry and Word Count (LIWC) [60]. Summaries were obtained for frequencies of emotion words, which were supplemented with a range of emoticons and Internet slang expressions for emotions. Social words and personal pronoun counts were also obtained. A word count for the target categories in the dictionary was extracted and these counts were uploaded to the server. This was repeated every 24 h to collect the posts that occured across the duration of *MoodPrism* use. The post content temporarily stored by *MoodPrism* was then deleted.

#### Experience Sampling

**Figure 1:**
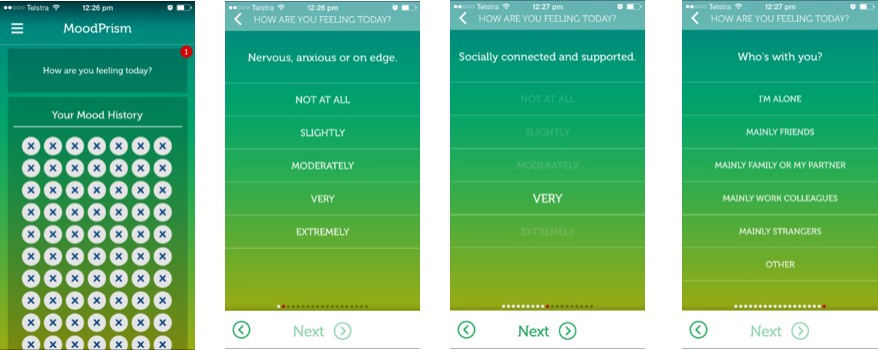
Screen shots from app showing experience sampling method.

*MoodPrism* utilized ESM to deliver a short set of questions to users daily (Figure 1). Prompts were delivered at a quasi-random time between user-defined hours (eg, 9:00 am-9:00 pm) for 30 days.

The questions captured a real-time assessment of the user’s emotional well-being, event-related experiences, and their context. Emotional state questions comprised 4 questions on psychological illhealth (depression and anxiety), 4 on emotional state (positive and negative affect, arousal, and control), and 4 on positive functioning (social connection, motivation, meaning, and self-esteem). Positive and negative event-related experiences were assessed by the type of event experienced and a rating of the event’s affective strength (from “slightly” to “extremely positive or negative”). The type of event was selected from a range of options drawn from stressor event questionnaires [61-65] and modified as a short list of the most common event domains (eg, school or work, physical health, material possessions, or social experience domain). Context was assessed via 2 questions, 1 for social context (who the user was with at the time of the report) and environmental context (where they were at the time of the report). Specific questions are given in Table 1.

In addition, a weekly prompt was delivered that requested a short voice recording to serve as an implicit measure of emotional state [51,53]. Users were prompted to read a standardized piece of text at the start and the end of the recording, and within that window to describe freely how they were feeling at that time.

#### Psychological Assessment Questionnaires

**Figure 2:**
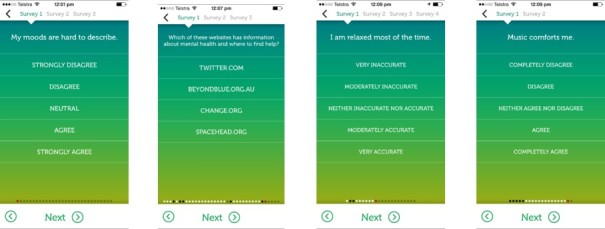
Screenshots showing examples of longer psychological questionnaires.

A number of questionnaires were available for completion at the onset of the app use, providing baseline measures of emotional well-being as well as data on potential moderators or confounding variables (see Figure 2). These questionnaires were categorized into survey “blocks” and displayed on the *MoodPrism* homescreen until their completion. This served to organize the questionnaires into manageable chunks for users to complete in their own time. A subset of these questionnaires was also delivered at the end of the month-long period to enable assessment of whether the app may have affected the well-being measures. A description of these questionnaires was provided in Table 2.

**Table 1:** Qualitative feedback: questions guiding qualitative feedback forums.

Broad question	Prompts
Was the app easy to use?	**Privacy issues (eg, social networking sites)** Was it clear to you why you were providing the information that you did? Why did you opt-in or opt-out of connecting your social media accounts? What things would be an incentive to opt-in?
	**Can you imagine anyone using the app without incentives?** Who do you think would benefit from using it? Was it clear to you that you were earning entries into a draw to win an iPad? Was it clear how the prize entries were being awarded? Did this consciously motivate you to use the app?
	Were the colors or emoticons used in the mood feedback helpful?
How did you find the daily prompts?	**Did they get in the way at all?** Were significant events captured? What kind of event did you feel was appropriate to report (major, minor, or both)?
How did you find the feedback?	**Mood feedback** Did you notice yourself paying more attention to the way you feel than usual? When you started using the app, was it made clear that reporting your mood could improve your mental health and well-being?
	**Surveys** Mental health info or contacts – did you explore any of these? Were they useful? Did you ever find the overview upsetting or negative?

**Table 2:** Sample feedback provided by beta-testers.

Theme	Sample responses
**Positive feedback**	
Aesthetically pleasant	It looks nice!
Easy to use	Seamless and smooth to use
Daily reports quick to complete	Simple set of responses takes only a few minutes daily – easy to use daily
Feedback useful and specific	Targeted questions give specific feedback about links between mood and daily activities Colored display of mood was useful representation [sic] Liked unlocking of content - motivated to keep using Feedback was not upsetting
Good to be able to get feedback about how feelings change daily	The ease of the app and being able to check in how exactly I’m feeling at a certain time
**Negative feedback**	
Wording of some questions confusing	Many questions in the introductory questionnaires are confusing double-negative repeats of previous questions, combined with putting negative responses near the top (where you expect positive ones) is confusing. I've never been irked when people expressed ideas very different from my own: “Yes or No”. Is it possible to put Agree or Disagree instead?
Some content can make you feel negative	Quite morbid things in the list of “most negative thing to happen to you today” -- makes me imagine some pretty terrible rare events like “death of a loved one”, etc. -- not a great thing to remind someone with depression to think about on a daily basis. / Many questions are quite negative like this -- you think about how stressed, worried, out of control, etc. you are -- creates a major disincentive to participating -- they're not things you want to dwell on when you're depressed.
Feedback clarity	The summary information for tracking well-being across times seems simplistic. For example, if I was in a good but deactivated mood, it said I was “on my way to thriving” - but of course it's not healthy to be highly activated ALL the time. The other thing I thought could be made clearer is what the numbers on the main screen mean - they're all different colors for the different days of the month but not sure what those numbers or colors mean
ESM functionality	There are a couple of categories I felt were missing when logging the things that happened today. On the “who are you with” screen, the option of “partner” would be useful. The “won something” category in the positive events screen was less useful. No positive event option for work
Privacy or information issues	Need trust in the app to give permission for social media sharing. So should give permission later on, perhaps after surveys, after built trust in app after some use Location information should be clarified to be postcode, not specific GPS point
Installation issues	Hard to download

#### Feedback

The final design feature of *MoodPrism* was the provision of a range of feedback to the user on their emotional well-being and mental health. This feedback was organized in consultation with the Australian mental health organizations *beyondblue* [66] and headspace [67], research literature on mental health and well-being, and expert advice on currently available mental health apps.

The feedback was available at several stages (see Multimedia Appendix 2):

- On the completion of a survey block, users were provided a summary of their general score on one of the surveys within that block.
- On completion of each daily report, users were provided with a color-coded brief description and custom emoticon representing their emotional state on that day. Weekly and monthly overviews were also available when multiple ESMs were completed.
- On completion of 1 week’s worth of ESMs, “positive mental health” data provided individualized feedback (based on their positive health responses), which included links to positive health websites and apps.
- On completion of 2 weeks’ worth of ESMs, depression and anxiety data were collated to provide individualized feedback on mental illness risk (based on their PHQ-4 responses). Recommendations and supporting links to mental health websites or contacts were also provided, as well as advice suitable to the user’s emotional functioning over the past 2 weeks.

### Database Security and Storage

With such extensive and potentially identifiable information being collected by *MoodPrism*, data storage and data security became a major priority. The following considerations were made regarding data storage in adherence with industry and University [68] standards, the *Privacy and Data Protection Act 2014*, and the *Guidelines for Ethical Practice in Psychological Research Online * as outlined by the British Psychological Society [69].

Immediately following the survey collection, data were stored on the user’s mobile phone prior to being uploaded encrypted into a secure database every 24 h. All data uploaded from the user’s phone was stored on an Amazon Web Services server. This database was protected by a firewall and regularly updated security protocols. The data stored were anonymized at the point of upload. All potentially identifiable information was removed from the data and only the device ID was retained (functioning as a randomly generated participant code). Data were only accessible online by authorized users via Secure Shell (SSH), which authenticates server access with digital certificates and encrypted passwords. All communication between authorized users and the server also occurred through HTTPS. This ensured that all information passed between the server and the researchers was encrypted and cannot be accessed or manipulated by a third party.

With regard to social media data, explicit consent to access Facebook or Twitter accounts (“opt-in”) was provided by the user. Their social media credentials were stored locally on the phone but were never uploaded to the server. All Facebook and Twitter posts’ content were processed locally in the mobile phone’s memory and aggregated word counts were generated. Only the aggregate word count was uploaded to the storage server.

## Results

The app was initially tested by both the researchers and the app developers for minor issues and bugs. A small convenience sample of independent, nonclinical users (N=11; age range=16-52 years; 4 males, 7 females) was then recruited to test the app to generate feedback on the functionality and usability of the app to the researchers and app developers. They used *MoodPrism* daily over a 30-day period and kept notes of their user experience. Information about the study was provided to the participants and electronic consent was required before the app could be used.

The test sample was invited to provide more intensive qualitative feedback by either Web-based questionnaire (n=5) or via attendance at a focus group session (n=6). Focus group participants also provided quantitative feedback by completing the Mobile Application Rating Scale (MARS) [70]. The MARS is a multidimensional measure for trialing and rating the quality of mobile phone apps, and has demonstrated interrater reliability and internal consistency. All beta-testers were also invited to discuss or provide emailed notes on their user experience. Broad questions were posed, and prompts were provided where necessary (see Table 1). (No attempt was made to analyze the emotional well-being data from the beta-testers, as the sample was small, and this aim was beyond the scope of the current paper, the primary aim of which was to provide information on the development of the app.)

Themes extracted from the comments provided via the focus group or Web-based feedback are presented in Table 2.

The testing of the app with this sample was approved by the Monash University Human Research Ethics Committee (Approval # CF14/968 – 2014000398). App development was completed in 2015 and tested over June-July 2015. The app was then revised in response to feedback received and the final version of the app prepared. The app was then released on the Google Play (Android) and Apple (iOS) stores. Future publications will report empirical data from this app, with the scope of the current publication limited to the development process only.

**Figure 3:**
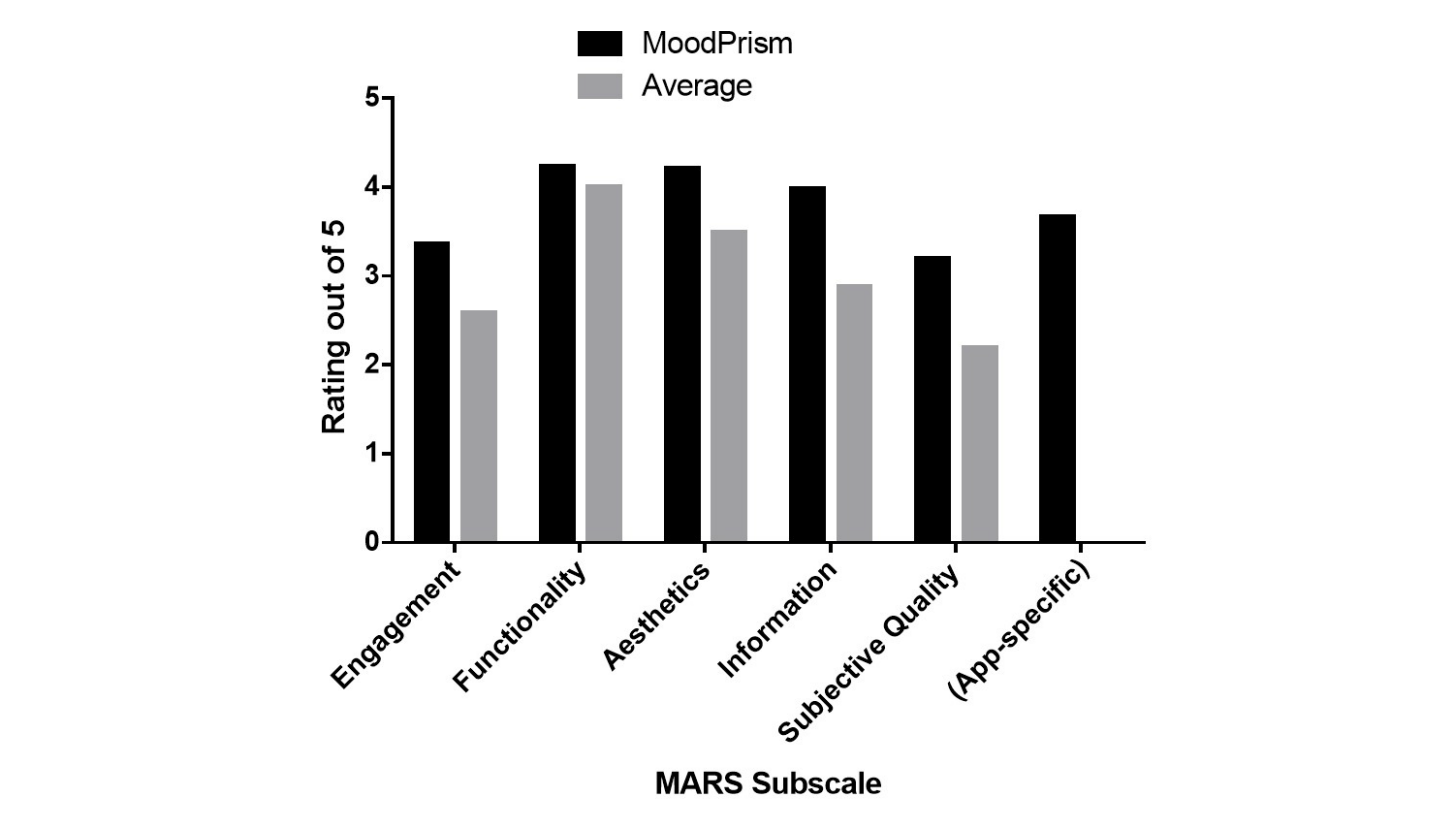
Quantitative feedback: beta-tester ratings on the Mobile Application Rating Scale (MARS) subscales (N=11).

Feedback about the functionality and usability of the app was obtained from 11 beta-testers, who completed a standard survey of app usability, the MARS. The results are presented in Figure 3.

MARS ratings for the *MoodPrism* app exceeded the average rating for 50 apps reviewed by Stoyanov et al [70] for each MARS subscale. Highest satisfaction ratings were obtained for items relating to the app’s graphics quality (eg, buttons, icons), gestural design (eg, swipes, scrolls), ease of use (eg, clear menus), credibility of the information sources, the layout aesthetics, and increased awareness of mood. Lowest ratings were obtained for entertainment value (eg, fun to use), customization options, likelihood to change behavior, motivations to address mood and interest, and likelihood to recommend to others.

The results from the focus group sessions and emailed responses from all 11 beta-testers are also summarized in Table 2.

The majority of issues identified by the beta-testers were addressed in the final version of the app. For instance, the order of positively or negatively worded options was made consistent across all questionnaires, additional information on how location and social networking data will be used was provided, with reassurance that information collected was deidentified was added, and an explanatory key was provided for interpreting colors and emoticons. The only issues that were not able to be addressed related to the integrity of psychometrically validated questionnaires (and therefore wording could not be altered), inclusion of negative content (which was important to the primary purpose of the app), or installation difficulties (as they related to the trial version only, and would not be present in the Apple and Android Web-based stores).

## Discussion

### Principal Findings

In this paper, we demonstrated how mobile phone technology could be harnessed to overcome several challenges in current mental health research and practices. Key needs we aimed to meet by developing this tool included the following: real-time monitoring of emotional functioning, assessing the full spectrum of emotional well-being, confidential access to mental health support and information when required, and to reduce obtrusiveness of regular monitoring.

*MoodPrism* was developed on both iOS and Android mobile phone platforms as an app to monitor emotional well-being in real time. It achieved this using ESM and collection of behavioral data via mobile phone apps (addressing challenge 1). It included assessment of daily positive psychological functioning (or “flourishing” [55]) in addition to more traditional assessment of negative psychological functioning (depression and anxiety) (addressing challenge 2). *MoodPrism* offered users a range of resources and links to enhance mental health literacy and access to professional mental health support, which vary depending on their current emotional functioning (addressing challenge 3). *MoodPrism* also incorporated voice monitoring, social networking site, and music playlist data collection as the first steps toward less obtrusive monitoring of emotional well-being for extended periods (addressing challenge 4)—although extensive algorithmic modeling will be necessary to achieve this goal. In sum, *MoodPrism* successfully responded to 4 key challenges in the emotional mental health domain. A number of important learnings were also achieved during this project, which may be helpful to outline for future researchers considering developing a mental health app [36].

### Considerations When Developing a Research-Based Mental Health App

Development of mental health apps is a relatively young field, and the guidelines to support researchers and app developers are not yet widespread. During the development of *MoodPrism*, a number of key issues were identified that could be helpful to researchers developing apps for mental health research and practice. These issues are briefly outlined in the following and then recommendations for consideration in future research are summarized in Figure 1.

First, it is important to recognize the different priorities of app developers and researchers (and mental health practitioners). For example, the *MoodPrism* researchers’ main goals were database integrity, psychometrically sound questionnaires, and ethical administration of sensitive content. The app developers’ main goals were an enjoyable user experience, good design, simple user interface, brief page content, and anonymous data storage. Identifying these goals and coming to an agreement on how they should be prioritized could help design an app that optimizes functionality (and therefore will be used by the participants) with integrity (so that the data are suitable for analysis). With *MoodPrism*, the researchers’ priority to maintain psychometric properties of questionnaires was in conflict with the app developers’ priority for good user interface and design. Administration of long questionnaires was overcome by creating brief checkpoints or “blocks” of surveys to complete, each with a portion of feedback provided as a reward to incentivize completion of long surveys. Similarly, the developers’ database priorities were guided by industry standards for data collection and storage. At times, this conflicted with the researchers’ need to obtain sufficient details; for example, anonymity of social media posts initially prevented the integrity of coding processes from being verified. Coding solutions were eventually achieved, but considerable delays could have been avoided if the database requirements were thoroughly discussed at the project’s outset. When these conflicting priorities were identified, a solution was often achieved that produced the unexpected benefit of optimizing outcomes for both stakeholders. For example, the chunking of questionnaires not only improved the user experience, but also was likely to improve the validity of data as participants were less likely to fatigue, or resort to nonserious responding.

Second, sufficient time should be quarantined at the outset for planning, and at the completion for beta testing and revision. App developers’ schedules can overlook the details involved in translating research requirements into the app space, and as a result underestimate the time involved. Database APIs for commercial apps also tend to have simpler output requirements than is often essential for advanced statistical analyses. A failure to identify the more complex necessities of the app’s function at the outset can result in over simplistic transition of features into the app, and subsequent delays in revision to meet research needs. Time spent presenting the entire app’s contents clearly up front to app developers will help avoid significant delays during development. Time should also be sufficient at the outset for complete storyboarding and wireframing of the app to ensure both parties agree on the app’s format and presentation. Aesthetics that work well in commercial apps do not always translate well for research content, which may out of a necessity include lengthier content or inflexible formatting or labeling of items (eg, traditional Likert-type scales in psychological questionnaires). Samples of similar app presentations that are known to work effectively with this type of content should if possible be reviewed and the best features identified. Allowing sufficient time for planning should also ensure that clear milestone dates are set, post which no further changes or additional content can be made by researchers or practitioners until trialing. Ongoing modifications can magnify delays for app developers and confuse versions being delivered. Sufficient time when the app is being finalized is also critical. Users should be allowed a sufficient trial period to allow testing of the app in various contexts, and the schedule should also ensure that they are able to report back both individually, and where possible as a part of group discussion. Focus groups are invaluable for identifying common themes across users, as well as allowing more singular experiences to emerge.

Third, communication among app developers and researchers or practitioners should be managed centrally. A flexible Web-based platform (such as “Basecamp”) provides project management tools such as discussion threads, allocation of tasks, a central file repository, and reminders. Progress of tasks should be monitored regularly and updates provided when item check off is delayed. Clear assignment of tasks avoids tasks being overlooked, and ensures accountability.

Fourth, methods to evaluate the app should be included within the app itself. Commercial apps can contain simple “thumbs up” or star ratings, but this is unlikely to be sufficiently informative for research or practitioner needs. Importantly, it is helpful to obtain assessments of the various aspects of the app, including commercial considerations such as aesthetics and functionality as well as those of central interest to researchers, such as ethics or trust and integrity. Published app assessment measures such as the MARS for health apps should be considered if possible. This will allow standardization and comparability across apps in the mental health space, and to build integrity and an evidence base for improvement of mental health apps over time.

**Figure 4:**
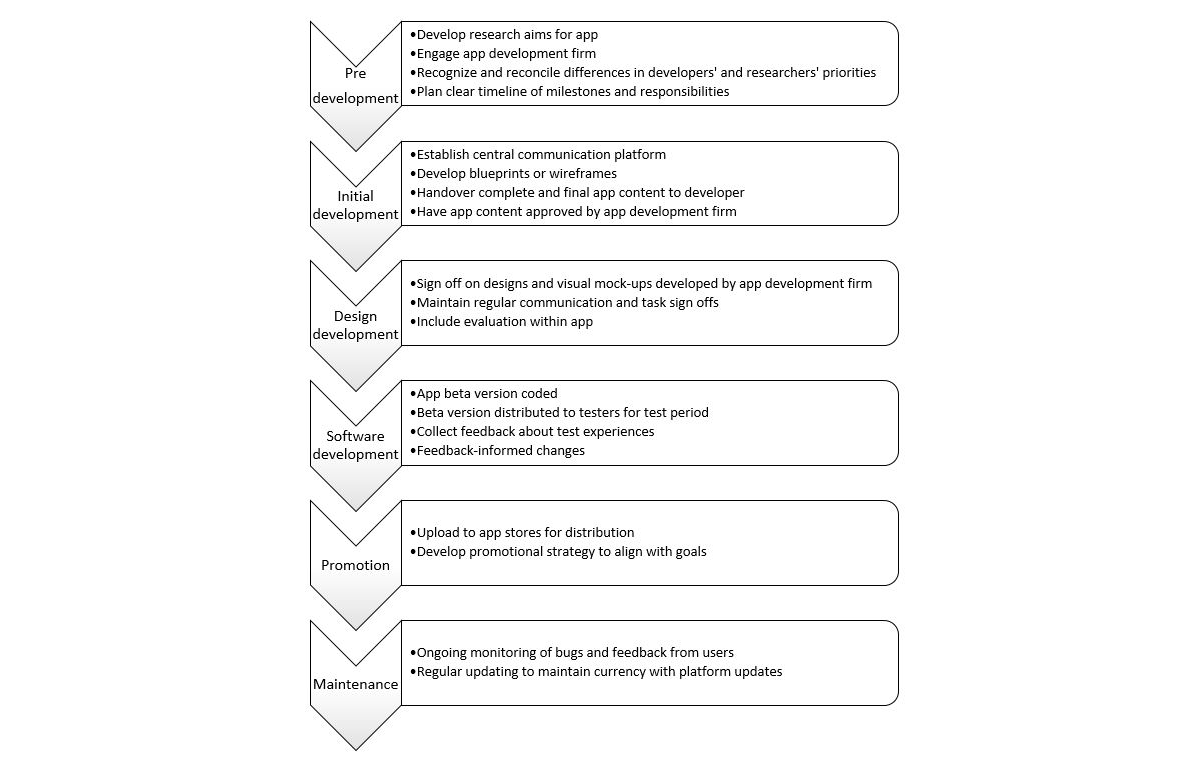
Recommended steps for researchers engaging in the app development process.

Our experiences researching and developing mental health apps have yielded a number of important practical insights of value to researchers in this field. The issues highlighted during the development of *MoodPrism*, taken together with our recommendations documented elsewhere [36], are summarized in Figure 4.

### Potential Applications of *MoodPrism* in Psychological Research

The development of a research mobile phone tool such as *MoodPrism* has enormous potential within the mental health field. Several applications of *MoodPrism* currently in progress are summarized in the following to illustrate the power of flexible, real-time monitoring using this platform.

#### Automated Prediction of Mental Health Risk

One of the most exciting promises for data-rich apps like *MoodPrism* is the development of algorithms which allow automated prediction of emotional health. This modeling could determine the minimum number of constructs required to reliably predict significant changes in emotional well-being, which could be used to inform a more streamlined and userfriendly app. Importantly, it is unlikely that any 1 or 2 variables will provide reliable prediction of such changes; a strength of *MoodPrism* is that it provides a breadth of variables that can be used to answer diverse and important research questions. Various algorithms may be identified, for instance, which discriminate between periods of stability and decline, and *MoodPrism* could then unobtrusively monitor for this change, and provide targeted mental health support to the user. This extends previous research that demonstrates feasibility of such modeling [40,41,71] by utilizing predictors already established in previous research to be associated with mental health (such as online social networking) rather than only those mobile phone sensors that are convenient to record (such as app use and activity).

#### Improving Emotional Self-Awareness, Mental Health Literacy, and Mental Health and Well-Being Outcomes

Bakker et al [36] detail how mental health apps can be categorized as reflection-, education-, or problem-focused. *MoodPrism* is largely a reflection-focused app aimed at improving a user’s emotional self-awareness by encouraging the user to report their thoughts, feelings, or behaviors and then reflect upon them. There is also an education component in *MoodPrism* that provides access to mental health information and resources. Use of this type of mental health app may therefore result in improvements in mental health and well-being. Kauer et al [72] found evidence that using a mobile phone app that promotes self-reflection through mood tracking can increase ESA and decrease depressive symptoms. Furthermore, rigorous study is needed to explore the mental health benefits of *MoodPrism* and other similar reflection-focused or education-focused apps, as very few randomized controlled trials have been conducted to investigate the efficacy of mental health apps [37]. Importantly, mobile phone technology complements traditional emotion monitoring techniques such as CBT-based recording worksheets [73,74], by increasing recording of subtle changes in behavior in real time. The innovative pairing of changes in emotional well-being with rapid delivery of mental health information has the potential to improve a user’s access to relevant resources such as Web-based health portals (eg, eheadspace, eHub), or local GPs when it is needed [75-77].

#### Leveraging Behavioral Data on Social Media to Gain Insight Into Mental Health and Social Context

Users of social networking sites leave rich digital traces of their social behavior, which includes the structure of their friendship networks and the written interactions between connections [78-80]. The quality of interactions on social network service (SNS) has been shown to hold important relationships with mental health. Positive interactions are associated with better mental health outcomes, and negative interactions may exacerbate mental illness [81-83]. However, how certain individual characteristics might lead a user to gain benefit or detriment from their SNS use is yet to be clearly described [84]. This requires access to both SNS data and the administration of psychometrically sound surveys to profile the users of SNSs. By profiling SNS users and better tapping into the interindividual variation in SNS use, the accuracy of SNS language models for mental health prediction could be improved [85] and some of the conflicting findings around the use of SNS and its mental health impact could be disentangled [85]. Furthermore, apps like *MoodPrism* enable SNS data to be associated in real time with ESM assessments of mood and psychological surveys. Time-sensitive linking of self-reported mood change and emotional expression in SNS posts may also provide evidence to support the use of SNS data and language analysis as a tool for mood and mental health tracking overtime.

#### Predicting Resilience Patterns to Everyday Significant Events

Event-based resilience research explores individual capacities to maintain healthy psychological functioning in response to naturally occurring stressor events [86,87]. Previous research methodologies use cross-sectionally designed studies and typically rely on retrospective reports [88-90]. These provide only partial snapshots of an individual’s capacity for resilient responding and can be subject to recall biases. The collection of *MoodPrism's* daily reports of psychological well-being, as well as the presence or absence of stressor events, is therefore pertinent to advancing event-based resilient research methodologies. Such methodological approaches allow for multiple snapshots in mood responding that, when compiled, create more representative, real-time observation of dynamic fluctuations that occur in an individual’s mood responses to stressor events. Such data will permit a more accurate exploration and identification of the heterogeneous mood trajectories that individuals display following stressor experiences [85,87,91-93]. Favorable patterns of responding, reflecting the maintenance of psychological functioning, can be identified and profiled to explore important factors that discriminate resilient individuals from other groups that reflect less-resilient patterns of responding.

### Conclusions

Development of mental health apps such as *MoodPrism* maximize health impact by harnessing the opportunities offered by mobile phone technology. Approximately, three quarters of the US and Australian populations own a mobile phone, and around 3 in 4 of those never leave home without their mobile device [31,94]. People check their mobile phones up to 150 times a day [30], demonstrating that mobile devices offer unprecedented access to everyday behavior. Incorporating evidence-based monitoring of emotional health into routine mobile phone apps can provide a powerful and flexible methodology for increasing personal control over one’s own emotional health. Capitalizing on inbuilt tools within mobile phones—such as music players, voice recorders, and social network media—to contribute data further enhances the potential of such apps to sensitively monitor emotional health over extended periods of time, while remaining unobtrusive. People (particularly young people) often find mobile phone technologies more engaging, anonymous, and less stigmatizing than other means of accessing help, and therefore are much more likely to use this methodology [16]. The new technologies described in this paper not only complement traditional approaches or educational tools supporting mental health but also have the potential to enhance their reach by overcoming many of the barriers currently challenging the reliable surveillance of emotional well-being.

## Appendix

Multimedia Appendix 1 Details on 3 forms of data (automatic, experience sampling, and psychological surveys) collected from *MoodPrism*.

Multimedia Appendix 2 Feedback generated by the subjects while using *MoodPrism*.

## Abbreviations

APIs: application programming interfaces
CBT: cognitive behavioral therapy
ESM: experience sampling methodology
LIWC: Linguistic Inquiry and Word Count
SSH: Secure shell
SNS: social network service
