# Supplementary material for: Development of a Mobile Phone App to Support Self-Monitoring of Emotional Well-Being: A Mental Health Digital Innovation
Source: JMIR Ment Health. 2016 Nov 23;3(4):e49. doi: 10.2196/mental.6202 (PMC5143469; doi:10.2196/mental.6202)
Supplement: Multimedia Appendix 1 [file mental_v3i4e49_app1.pdf]

Table 1. Details on 3 forms of data (Automatic, Experience sampling, and Psychological surveys) collected from MoodPrism.

Automated data collection		Experience sampling items		Psychological questionnaires		
**Source**	**Data (downloaded once every 24 h, subject to user permissions)**	**Target and questions**	**Source**	**Questionnaire**	**Purpose**	**Source**
Facebook	For each post in history (up to last 50):<br>- Date and time of post<br>- Length of message (characters)<br>- Number of words in message<br>- Number of positive, negative, self-pronoun, other-pronoun words<br>- Number of likes<br>- Number of comments<br>- Number of tags<br>- City and postcode (if available) of where posted	**Emotional well-being**<br>How were you feeling just before you were prompted by this app? (rated on a 5-point scale from "not at all" to "extremely")<br>**1.** Nervous, anxious, or on edge<br>**2.** Not able to stop or control worrying<br>**3.** Little interest or pleasure doing things<br>**4.** Feeling down, depressed, or hopeless<br>**5.** Active or alert<br>**6.** Negative or unpleasant<br>**7.** Positive or pleasant<br>**8.** In control of what I'm doing<br>**9.** Socially connected and supported<br>**10.** Motivated, engaged, and interested<br>**11.** Life is meaningful and with purpose<br>**12.** Feeling good about myself	Drawn from PHQ-4 [59];<br>3 dimensional models of affect [60,61];<br>The WHO[a] emotional well-being scales (WHO-5) [62]; positive health literature [55]; single item self-esteem measures [63]	**At baseline only**		
				Demographics	Sample description or Potential moderator or confounding variable	Custom developed
				Life event scale	Potential moderator or confounding variable	[69]
				Multidimensional scale of perceived social support	Potential moderator or confounding variable	[70]
				Social desirability scale	Assessment of reliable responding	[71]
				IPIP	Potential moderator or confounding variable	[72]
				Rosenberg's self-esteem	Potential moderator or confounding variable	[73]
				Barcelona music rewards scale	Potential moderator or confounding variable	[74]
				Technology use survey	Potential moderator or confounding variable	Custom developed except for 1 item drawn from [75]
				**At baseline and 1 month follow up**		

Twitter<br><br>Music library	For each tweet in timeline (up to last 50):<br>- Date and time of tweet<br>- Tweet client site or software or app used<br>- Length of message (characters)<br>- Number of words in message<br>- Number of positive, negative, self-pronoun, other-pronoun words<br>- Number of retweets<br>- Number of favourites<br><br>For each song in the user's music library<br>- Song Title<br>- Genre<br>- Artist<br>- Album<br>- Last Played<br>- Play count<br>- Rating<br>- Release date<br>- Duration	**Event-related experiences**<br><br>What's the most positive thing that's happened to you in the past 24 h?<br>• Nothing positive happened<br>• Positive social experience (with friends, family, strangers, etc)<br>• Obtained material item (bought or won something, received a gift, etc)<br>• Positive experience at work or school<br>• Positive experience outside of work or school<br>• Positive health or fitness experience<br>• A happy occasion (e.g., birthday, wedding, holiday)<br>• Other (with option to type in detail)<br>What's the most negative thing that's	Items modified from various stressor event questionnaires [64-68]	Emotional self-awareness scale	Evaluation of emotional awareness change	[76]
				Warwick Edinburg well-being scale	Evaluation of well-being change	[77]
				PHQ-9[c]	Evaluation of well-being change	[78]
				GAD-7[d]	Evaluation of well-being change	[79]
				Mental health literacy questionnaire	Evaluation of mental health literacy change	Vignettes adapted from [80]<br>Additional questions drawn from *beyond blue* website.
				Brief resilience scales	Evaluation of mental health literacy change	[81]
				Coping self-efficacy scale	Evaluation of mental health literacy change	[82]
				**At 1 month follow up only**		

		happened to you in the past 24 hours?<br>• Nothing negative happened<br>• Negative social experience (with family, friends, strangers, etc)<br>• Loss of valued material item (misplaced, theft, etc)<br>• Negative experience at school or work<br>• Negative experience outside of work or school<br>• Personal health problems (illness, injury, etc)<br>• Health problems of someone close to you (illness, injury, death, etc)<br>• Other (with option to type in detail)<br>Followed by options for all (except "nothing"):<br>How positive or negative was it?<br>• Slightly<br>• Moderately		Feedback questionnaire	Assessment of app quality	Custom developed (although broadly consistent with factors of MARS[b]).

		• Very<br>• Extremely<br><br>**Context**<br><br>Where are you? (drop-down selections)<br>• At home<br>• At someone else’s place<br>• At work, uni, or school<br>• At a leisure venue (eg, cinema, shops, park, sporting venue)<br>• Travelling or commuting<br>• Other (with option to type in detail)<br>Who’s with you? (Drop-down selections)<br>• I’m alone<br>• Mainly friends<br>• Mainly family or my partner<br>• Mainly work colleagues<br>• Mainly strangers<br><br>Other				

[a]WHO: World Health Organization.

[b]MARS: Mobile Application Rating Scale.

[c]PHQ-9: Patient Health Questionnaire-9

[d]GAD-7: General Anxiety Disorder scale-7

55. Keyes, C.L., Mental illness and/or mental health? Investigating axioms of the complete state model of health. Journal of Consulting and Clinical Psychology 2005;73(3): p. 539-548.
59. Kroenke, K., et al., An ultra-brief screening scale for anxiety and depression: the PHQ-4. Psychosomatics 2009;50(6): p. 613-21.
60. Mehrabian, A., Russell, J.A. An approach to environmental psychology (1 ed.). Cambridge, Mass.: MIT Press, 1974.
61. Russell, J. A. A circumplex model of affect. Journal of Personality and Social Psychology 1980;39:1161–1178.
62. Bech, P., O.R. Kjoller, and N.K. Rasmussen, Measuring well-being rather than the absence of distress symptoms: a comparison of the SF-36 Mental Health subscale and the WHO-Five Well-Being Scale. International J Methods Psychiatr Res, 2003. 12(2): p. 85-91.
63. Robins, R. W., Hendin, H. M., Trzesniewski, K. H. Measuring global self-esteem: Construct validation of a single-item measure and the Rosenberg Self-Esteem Scale. Personality and Social Psychology Bulletin 2001;27:151-161.
64. Cheng, C. Assessment of major life events for Hong Kong adolescents: The Chinese adolescent life event scale. American Journal of Community Psychology 1997;25(1):17-33.
65. Coddington, R. D. The significance of life events as etiologic factors in the diseases of children—II. A study of a normal population. Journal of Psychosomatic Research 1972; 16(3):205-213.
66. Newcomb, M. D., Huba, G. J., Bentler, P. M. A multidimensional assessment of stressful life events among adolescents: Derivation and correlates. Journal of Health and Social Behavior 1981;22:400-415.
67. Swearingen, E. M., Cohen, L. H. Measurement of adolescents' life events: The junior high life experiences survey. American Journal of Community Psychology 1985;13(1):69-85.
68. Waaktaar, T., Borge, A. I. H., Fundingsrud, H. P., Christie, H. J., Torgersen, S. The role of stressful life events in the development of depressive symptoms in adolescence—A longitudinal community study. Journal of adolescence 2004;27(2):153-163.
69. Holmes, T.H., Rahe, R.H. The Social Readjustment Rating Scale. J Psychosom Res 1967;11(2): 213–8
70. Zimet, G.D., et al., The Multidimensional Scale of Perceived Social Support. Journal of Personality Assessment 1988;52(1):30-41.
71. Reynolds, W.M. Development of reliable and valid short forms of the Marlowe-Crowne social desirability scale. Journal of Clinical Psychology 2006;38(1):119-125.
72. Donnellan, M.B., Oswald, F., Baird, B., Lucas, R.E., et al The Mini-IPIP Scales: Tiny-Yet-Effective Measures of the Big Five Factors of Personality. Psychological Assessment, 2006 ;18(2), 192-203
73. Rosenberg, M. Society and the adolescent self-image. Princeton, NJ: Princeton University Press, 1965.
74. Mas-Herrero, E. et al. Individual differences in music reward experiences. Music Perception 2013; 31(2):118-138.
75. Young and Well Cooperative Research Centre. 2016-06-14. URL:http://www.youngandwellcrc.org.au (Accessed: 2016-06-14. (Archived by WebCite® at http://www.webcitation.org/6iGyrSPAQ)
76. Kauer, S.D. et al. Self-monitoring Using Mobile Phones in the Early Stages of Adolescent Depression: Randomized Controlled Trial. Journal of Medical Internet Research 2012;14(3):e67

77. Tennant, R. et al The Warwick-Edinburgh Mental Well-being Scale (WEMWBS): Ddevelopment and UK validation. Health and Quality of Life Outcomes 2007;5:63
78. Kroenke K, Spitzer RL, Williams JBW. The PHQ-9: Validity of a brief depression severity measure. J Gen Intern Med 2001;16: 606-613.
79. Spitzer RL, Kroenke K, Williams JBW, Löwe B. A brief measure for assessing generalized anxiety disorder: the GAD-7. Arch Intern Med 2006;166:1092-1097.
80. Reavley, N.J., Morgan, A.J, Jorm, F. F., Development of scales to assess mental health literacy relating to recognition of and interventions for depression, anxiety disorders and schizophrenia/psychosis. Australian and New Zealand Journal of Psychiatry 2014; 48(1):61-9.
81. Smith, B.W. et al The Brief Resilience Scale: Assessing the ability to bounce back. International Journal of Behavioral Medicine 2008;15:194-200.
82. Chesney, M.A., et al. A validity and reliability study of the coping self-efficacy scale. Br J Health Psychol 2006;11(3): 421-437
