# Supplementary material for: Development of a Mobile Phone App to Support Self-Monitoring of Emotional Well-Being: A Mental Health Digital Innovation
Source: JMIR Ment Health. 2016 Nov 23;3(4):e49. doi: 10.2196/mental.6202 (PMC5143469; doi:10.2196/mental.6202)
Supplement: Multimedia Appendix 2 [file mental_v3i4e49_app2.pdf]

Feedback type	Trigger	Scoring	Sample
**Psychological surveys**			
Normative feedback	When block of surveys completed	Scoring based on published guidelines, and feedback based on published normative data).<br>For example:<br>(<45) in the lower range of positive health scores (less than 75% of people)<br>(45-50) on the lower end of the average range of positive health scores (less than 50% of people)<br>(51-56) on the higher end of the average range of positive health scores (more than 50% of people)<br>(>56) in the higher range of positive health scores (more than 75% of people)	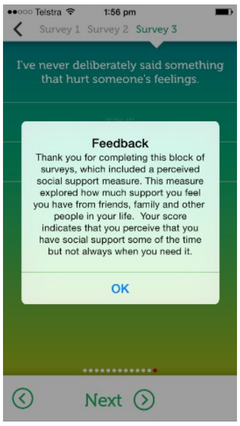
**Risk assessment**			

<table>
<tr><td>Prompt to seek mental health support</td><td>Red flag high score</td><td>PHQ or GAD score above 15 (as per published recommendations)</td><td>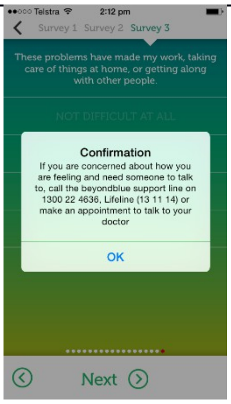
</td></tr>
</table>

Experience sampling self-reports

Visual (icon, colour) and descriptions of emotional state, as well as context information. Reported either in detailed (1 day), brief form (weekly), or overview (complete log) format.	On user request from day 1.	Based on 2-dimensional (arousal and valence) circumplex model of emotion (see below); color coding based on subjective convention.<br>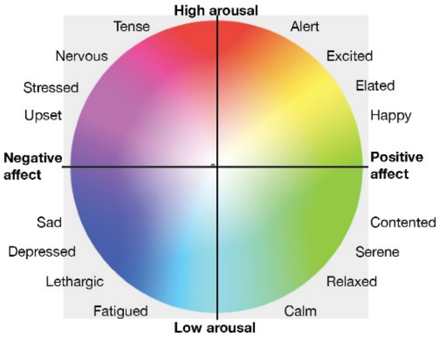	Overview:<br>Weekly view:<br>Daily detail:

Description of positive health function	On user request unlocked from day 8.	Scoring based on the sum of ESM items reflecting feelings of: positive, control, social connection or support, motivation or engagement, and meaning or purpose<br>• 5-10: Low score<br>• 11-19: Medium score<br>• 20-25: High score<br><br>Further information link options (rotating over time) include Smiling Mind, Healthy Habits, and Buddhify apps, and [well-being websites such as ”Authentic Happiness” and “Soul pancake”]()	
Description of depression or anxiety levels	On user request unlocked from day 15.	Scoring based on PHQ or GAD frequency of behaviors over a 2-week period (none, less than half the days, around half the days, every or most of	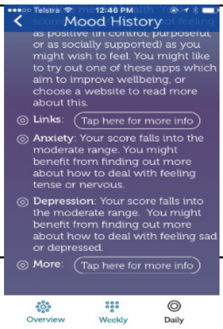

		the days)<br><br>Summed to produce<br>• 0-2: low<br>• 3-4: moderate<br>• 5-6: high<br><br>Further information link options (rotating over time) include
Progress toward entries into prize draw	Frequency counts and countdowns	**Day 1-7**: Counts down to unlocking further mood feedback (positive functioning feedback)<br>**Day 8-14**: Counts down to unlocking further mood feedback (depression or anxiety feedback)<br>**Every day**: Counts up number of days completed to yield number of entries into prize-draw.
